# Prevalence of Psoriatic Arthritis and Its Risk Factors Among Patients With Psoriasis in a Tertiary Care Center in Saudi Arabia

**DOI:** 10.7759/cureus.82782

**Published:** 2025-04-22

**Authors:** Raham A Alamri, Bashaer H Almahdi, Siham Marghalani

**Affiliations:** 1 Department of Dermatology, King Abdul Aziz Medical City, Jeddah, SAU

**Keywords:** autoimmune disease, cardiovascular comorbidities, prevalence study, psoriasis, psoriatic arthritis, psychiatric comorbidities

## Abstract

Introduction

Psoriatic arthritis (PsA) is a chronic inflammatory arthritis that develops in a subset of psoriasis patients, often leading to functional impairment and comorbid conditions. Early detection and management of PsA are essential for improving patient outcomes. This study aimed to (1) determine PsA prevalence among psoriasis patients at a tertiary care center in Jeddah, Saudi Arabia; (2) identify risk factors for PsA development; and (3) describe clinical PsA subtypes.

Methods

In this retrospective cross-sectional study, 414 psoriasis patients were evaluated using electronic medical records and supplementary telephone interviews. A nested case-control analysis compared PsA cases (n=53) to psoriasis-only controls (n=354). Descriptive statistics summarized prevalence and subtype distribution, while logistic regression assessed associations between PsA and potential risk factors.

Results

PsA prevalence in our population was 13%, with a female predominance, 36 (67.9%). Asymmetric oligoarticular arthritis, 20 (39.2%), was the most frequent subtype, followed by symmetric polyarthritis, 15 (29.4%). Nail involvement was present in 25 (52.1%) of PsA patients. Cardiovascular (47.2% vs. 35.6%) and psychiatric comorbidities (11.3% vs. 5.1%) were more common in PsA patients. Logistic regression identified family history of PsA (OR = 7.8; 95% CI: 1.44-42.2) and psychiatric comorbidities (OR = 4.5; 95% CI: 1.17-17.04) as significant predictors of PsA.

Conclusion

PsA affects a notable proportion of psoriasis patients and is associated with higher rates of cardiovascular and psychiatric comorbidities. Asymmetric oligoarthritis predominated, and family history emerged as a strong predictor. These findings underscore the need for early screening and multidisciplinary management. Larger multicenter studies are warranted to validate these associations.

## Introduction

Psoriasis is a chronic immune-mediated skin condition affecting approximately 2-3% of the global population, with varying prevalence across different regions [1]. It is characterized by scaly, erythematous plaques and can significantly impact quality of life. Among patients with psoriasis, many develop psoriatic arthritis (PsA), a chronic inflammatory disease involving the peripheral joints, axial skeleton, and entheses. While the exact etiology of PsA is not fully understood, genetic, immunologic, and environmental factors are thought to contribute to the inflammatory process. PsA patients present with joint pain and stiffness that worsens with prolonged immobility. The articular manifestations are varied and may shift from one pattern to another over time. PsA may precede, co-occur with, or follow the skin manifestations. However, only 13-17% of patients develop arthritis before the onset of psoriasis [2].

Global prevalence estimates of PsA vary considerably. A meta-analysis published in 2018 reported the prevalence of PsA among psoriasis patients to be 22.7% in European patients, 21.5% in South American patients, 19.5% in North American patients, 15.5% in African patients, and 14.0% in Asian patients [3]. Geographical variation can be explained by differences in HLA-B27 allele expression, methodological approaches, and underdiagnosis [4]. One meta-analysis reported the prevalence of undiagnosed PsA to be as high as 15.5% among psoriasis patients [5]. Diagnostic delay, which is associated with worse clinical and radiographic outcomes, has been reported to be 3.67 ± 6.42 years in Saudi Arabia [6]. In addition, PsA is linked to several cardiovascular and psychosocial comorbidities, including coronary artery disease, increased risk of death from cardiovascular events, depression, negative body image, sleep disturbances, and reduced work productivity [7,8]. These consequences further emphasize the importance of early detection in managing PsA. Although one study in Saudi Arabia detected higher incidence rates of subclinical synovitis and enthesitis in psoriasis patients compared to global reports, limited studies have been published on the prevalence of PsA in Saudi Arabia [9].

Once considered a mild disease, PsA is now recognized as a potentially debilitating condition that requires targeted treatment with continuous monitoring and follow-up. This study aims to determine the prevalence of psoriatic arthritis among patients with psoriasis and to identify associated risk factors and common clinical patterns in a tertiary care center in Jeddah, Saudi Arabia. Conducting our own epidemiological studies can take us one step closer to understanding PsA and enhancing treatment outcomes by tailoring care to vulnerable populations.

## Materials and methods

Study setting, study population, and study design

This study was conducted at King Abdulaziz Medical City, a tertiary healthcare center located in Jeddah, Saudi Arabia. The study included patients aged seven years or older who were diagnosed with psoriasis between January 1, 2019, and October 31, 2024. Patient identification was based on the International Classification of Diseases, Tenth Revision, Clinical Modification (ICD-10-CM) code L40. In total, 414 psoriasis patients were included. Additionally, PsA diagnosis was based on ICD-10-CM code L40.5 and documentation by dermatologists or rheumatologists in the medical records.

This is a cross-sectional study based on a retrospective review of electronic medical records. Relevant demographic and clinical data were collected from medical records, including age, sex, body mass index (BMI), psoriasis subtype, and comorbidities. In cases where essential data were missing from the medical records, patients were contacted via phone to complete the data collection. Furthermore, comorbid conditions were identified using corresponding ICD-10-CM codes, including hypertension (I10, U82.3), hyperlipidemia (E78), type 2 diabetes mellitus (E11, E13), depression (F33, U79.3), and anxiety (F41), along with documented diagnosis by physicians in the medical records.

To explore risk factors associated with the development of PsA, a case-control design was nested within the cross-sectional study. Patients with a confirmed diagnosis of PsA were assigned to the case group, while psoriasis patients without PsA formed the control group. Variables assessed as potential risk factors included age, sex, family history of psoriasis, history of infection, mechanical stress, and exposure to smoking or secondhand smoking.

Statistical analysis

Statistical analysis was performed using IBM SPSS Statistics for Windows, Version 27 (Released 2020; IBM Corp., Armonk, New York). A p-value < 0.05 was considered statistically significant, and a 95% confidence interval (CI) was applied to all analyses. Descriptive statistics were used to report frequencies, percentages, means, and standard deviations (SD) for patient demographics, PsA subtypes, comorbidities, and risk factors. Cardiovascular comorbidities (hypertension, hyperlipidemia, type 2 diabetes mellitus, obesity, and smoking exposure) and psychiatric comorbidities (depression and anxiety) were grouped for analysis. Chi-square tests and independent samples t-tests were used for univariate analysis to compare patients with and without PsA. Relative risks and odds ratios (ORs) were calculated to estimate associations. Binary logistic regression was employed to identify independent predictors of PsA, using variables such as age, sex, family history of psoriasis, mechanical stress, infection, and grouped comorbidities. A full model was compared to a constant-only model to evaluate the predictive value of each variable.

## Results

Study population and demographics

This study included 414 psoriasis patients with a mean age of 42 years (SD = 17.9). The majority of participants were female, 218 (52.7%). Among them, 53 (13%) were diagnosed with PsA, while 354 (87%) had no joint involvement. A family history of psoriasis was reported by 112 (35.0%) patients, whereas 9 (4.4%) reported a family history of PsA. The most prevalent psoriasis subtype was plaque psoriasis, 347 (85.3%), followed by guttate psoriasis, 38 (9.3%), pustular, 9 (2.2%), inverse, 9 (2.2%), and erythrodermic, 4 (1.0%) (Table 1).

**Table 1:** Demographic and clinical characteristics of psoriasis patients

Variable	Category	Frequency (%)	Mean (SD)
Age in years	-	-	42 (17.9)
Gender (N=414)	Male	196 (47.3%)	-
	Female	218 (52.7%)	
Family history of psoriasis (N=320)	Yes	112 (35%)	1.65 (0.48)
	No	208 (65%)	
Psoriasis subtype (N=407)	Plaque	347 (85.3%)	1.49 (1.25)
	Erythrodermic	4 (1%)	
	Pustular	9 (2.2%)	
	Inverse	9 (2.2%)	
	Guattate	38 (9.3%)	
Nail involvement (N=406)	Yes	153 (37.7%)	1.62 (0.49)
	No	253 (62.3%)	
Psoriatic arthritis (N=407)	Yes	53 (13%)	1.87 (0.340)
	No	354 (87%)	
Family history of psoriatic arthritis (N=206)	Yes	9 (4.4%)	1.96 (0.21)
	No	197(95.6%)	

PsA prevalence and patients’ characteristics

PsA was identified in 53 (13%) of psoriasis patients (Table 1). The mean age of PsA patients was 46.8 years (SD = 17.5), and the majority were female, 36 (67.9%). A family history of psoriasis was reported in 12 (30.0%) patients, while a family history of PsA was documented in 5 (20.8%). The most common clinical subtype of psoriasis among PsA patients was plaque psoriasis, observed in 42 (85.7%), followed by inverse psoriasis, 3 (6.1%), and guttate psoriasis, 3 (6.1%). Nail involvement was present in 25 (52.1%) patients. Regarding joint involvement, the most frequent PsA subtype was asymmetric oligoarticular arthritis, 20 (39.2%), followed by symmetric polyarthritis, 15 (29.4%), and distal interphalangeal (DIP) predominant arthritis, 11 (21.6%). Less frequent patterns included spondyloarthritis, 2 (3.9%), and arthritis mutilans, 2 (3.9%) (Table 2).

**Table 2:** Prevalence and characteristics of psoriatic arthritis in psoriasis patients

Variable	Category	Frequency (%)	Mean (SD)
Age in years	-	-	46.8 (17.5)
Gender (N=53)	Male	17 (32.1%)	1.68 (0.47)
	Female	36 (67.9%)	
Family history of psoriasis (N=40)	Yes	12 (30%)	1.7 (0.46)
	No	28(70%)	
Psoriasis subtype (N=49)	Plaque	42 (85.7%)	1.45 (1.2)
	Erythrodermic	1 (2%)	
	Pustular	0 (0.0%)	
	Inverse	3 (6.1%)	
	Guattate	3 (6.1%)	
Nail involvement (N=48)	Yes	25 (52.1%)	1.48 (0.51)
	No	23 (47.9%)	
Family history of psoriatic arthritis (N=24)	Yes	5 (20.8%)	1.79 (0.42)
	No	19 (79.2%)	
Psoriatic arthritis subtype (N=51)	Spondylarthritis	2 (3.9%)	3.76 (1.03)
	Arthritis mutilants	2 (3.9%)	
	Symmetric polyarthritis	15 (29.4%)	
	Asymmetric oligoarticular	20 (39.2%)	
	Distal interphalangeal predominant	11 (21.6%)	
	Polyarticular arthritis	1 (2%)	

Cardiovascular comorbidities in PsA patients compared to psoriasis-only patients

Cardiovascular comorbidities, including hypertension, type 2 diabetes mellitus, hyperlipidemia, obesity, and smoking or secondhand smoke exposure, were present in 25 (47.2%) of PsA patients compared to 126 (35.6%) of those with psoriasis alone. Among PsA patients, the most prevalent comorbidities were type 2 diabetes mellitus and hypertension, each reported in 16 (30.2%) patients. In contrast, among psoriasis patients without joint involvement, type 2 diabetes mellitus was the most common comorbidity, affecting 83 (23.4%) patients. Despite the higher prevalence in the PsA group, no statistically significant association was observed between cardiovascular comorbidities and PsA (p = 0.104; OR = 1.62; 95% CI: 0.90-2.89). Notably, the mean BMI was similar between the two groups (29 kg/m²) (Tables 3, 4).

**Table 3:** Comparison of cardiovascular comorbidities between psoriatic arthritis and psoriasis-only patients

Variables	Cardiovascular comorbidities among psoriasis patients with psoriatic arthritis, N=53		Cardiovascular comorbidities among psoriasis patients only, N=354	
	Frequency (%)	Mean (SD)	Frequency (%)	Mean (SD)
Overall	25 (47.2%)	1.52 (0.50)	126 (35.6%)	1.64 (0.40)
Hypertension	16 (30.2%)	1.70 (0.46)	68 (19.2%)	1.81 (0.39 )
Hyperlipidemia	12 (22.6%)	1.77 (0.42)	46 (13%)	1.9 (0.34)
Type 2 diabetes mellitus	16 (30.2%)	1.70 (0.46)	83 (23.4%)	1.77 (0.42)
Smoking or secondhand smoking	5 (9.4%)	1.91 (0.30)	24 (6.8%)	1.93 (0.25)
BMI	-	29.79 (6.94)	-	29 (7.96)

**Table 4:** Association between cardiovascular comorbidities and psoriatic arthritis The statistical test used for the analysis was the chi-square test for association and risk estimation. χ² = 2.65; degrees of freedom (df) = 1

Cardiovascular comorbidities	Psoriatic arthritis patients, N=53	Psoriasis-only patients, N=354	P-value	Odds ratio (95% CI)
Patients with cardiovascular comorbidities	25 (47.2%)	126 (35.6%)	0.104	1.62 (0.90–2.89)
Patients without cardiovascular comorbidities	28 (52.8% )	228 (64.4%)		

Psychiatric comorbidities in PsA patients compared to psoriasis-only patients

Psychiatric comorbidities, particularly depression and anxiety, were more prevalent among PsA patients, 6 (11.3%), compared to those with psoriasis alone, 18 (5.1%). Depression was the predominant psychiatric condition in both groups, affecting 5 (9.4%) of PsA patients and 11 (3.1%) of psoriasis-only patients. Anxiety was present in 2 (3.8%) of PsA patients and 8 (2.3%) of psoriasis-only patients (Table 5).

**Table 5:** Prevalence of psychiatric comorbidities in psoriatic arthritis and psoriasis-only patients

Variable	Psychiatric comorbidities among psoriatic arthritis patients, N=53 (n (%))	Psychiatric comorbidities among psoriasis-only patients, N=354 (n (%))
Overall	6 (11.3%)	18 (5.1%)
Depression	5 (9.4%)	11 (3.1%)
Anxiety	2 (3.8%)	8 (2.3%)

As shown in Table 6, depression was more common in PsA patients, 5 (9.4%), than in psoriasis-only patients, 11 (3.1%) (p = 0.044, Fisher's exact test). Although statistically significant, the association was weak (Cramér's V = 0.1). PsA patients had more than threefold higher odds of depression (OR = 3.25; 95% CI: 1.08-9.75), suggesting a potential link between PsA and increased depression risk. In contrast, anxiety did not show a statistically significant association with PsA (OR = 0.65; 95% CI: 0.14-3.16; p = 0.64) (Table 7).

**Table 6:** Association between depression and psoriatic arthritis The statistical test used for the analysis was the chi-square test for association and risk estimation. *Fisher’s exact p-value.

Depression diagnosis	Psoriatic arthritis patients, N=53	Psoriasis-only patients, N=354	P-value	Cramer’s V	Odds ratio (95% CI)
Depression	5 (9.4%)	11 (3.1%)	0.044	0.11*	3.25 (1.08–9.75)
No depression	48 (90.6%)	343 (96.9%)			

**Table 7:** Association between anxiety and psoriatic arthritis The statistical test used for the analysis was the chi-square test for association and risk estimation. *Fisher’s exact p-value.

Anxiety diagnosis	Psoriatic arthritis patients, N=53	Psoriasis-only patients, N=354	P-value	Odds ratio (95% CI)
Patients with anxiety	2 (3.8%)	8 (2.3%)	0.64*	0.65 (0.14–3.16)
Patients without anxiety	51 (96.2%)	346 (97.7%)		

Predictors of PsA

A logistic regression analysis was conducted, through a nested case-control design, to predict PsA using family history of PsA and psychiatric comorbidities as predictors (Table 8). The model was statistically significant (X² = 8.54, p = 0.014, df = 2). Patients with a family history of PsA were 7.8 times more likely to develop the condition (OR = 7.8; 95% CI: 1.44-42.2), and those with psychiatric comorbidities were 4.5 times more likely (OR = 4.5; 95% CI: 1.17-17.04), when controlling for the other variable. Other variables, such as age, cardiovascular comorbidities, BMI, history of mechanical stress, and history of infection, were initially included, but the backward LR model removed them at different steps due to lack of significance. The final model equation was:

Psoriatic arthritis = −2.05 + 2.05 (family history of psoriatic arthritis) + 1.5 (psychiatric comorbidities) (Table 8).

**Table 8:** Predictors of psoriatic arthritis based on logistic regression analysis Chi square = 8.54, p-value = 0.014. Psoriatic arthritis = -2.05 + 2.05 (family history of psoriatic arthritis) + 1.5 (psychiatric comorbidities).

Risk factors	Value	P-value	Odds ratio	95% CI
Family history of psoriatic arthritis (Reference = 1)	2.05	0.017	7.8	1.44–42.2
Psychiatric comorbidities (overall) (Reference = 1)	1.5	0.029	4.5	1.17–17.04

## Discussion

This study identified a 13% prevalence of PsA among psoriasis patients at a tertiary center in the western region of Saudi Arabia. This figure is consistent with global estimates but remains lower than the prevalence reported in Europe (22.7%) and North America (19.5%), while closely aligning with the Asian prevalence of 14% [3]. Various factors contribute to the variability of PsA prevalence, including genetic predisposition, access to healthcare, and the potential for underdiagnosis [4]. Additionally, methodological differences in research, such as cross-sectional study designs and variability in the selection of PsA populations, may further influence these estimates. Early detection of PsA is crucial in preventing long-term joint damage and improving the overall quality of life for affected individuals; therefore, conducting localized epidemiological studies can provide valuable insights into PsA and enhance treatment outcomes by tailoring care to vulnerable populations.

In our study, nail involvement was observed in 52.1% of PsA patients, reinforcing its recognition as a common feature of PsA. This aligns with prior research demonstrating a strong link between nail psoriasis and distal interphalangeal (DIP) joint disease. For instance, a cross-sectional study of 45 PsA patients reported that 64.4% had nail psoriasis, with regression analysis identifying nail disease as the most significant predictor of DIP arthritis (OR = 9.7, p = 0.05). Notably, 59.6% of digits with radiological DIP changes had concurrent nail involvement, and specific subtypes (onycholysis and crumbling) showed significant associations (p = 0.001) [10]. The mechanistic link may involve shared embryologic origins of the nail and DIP joint, with localized inflammation driving both pathologies. Future longitudinal studies should explore whether early nail changes predict progressive joint damage, which could guide targeted monitoring and therapy.

Initially, asymmetric oligoarticular PsA was believed to be the most common subtype, accounting for 70% of all cases [11]. However, more recent literature indicates significant variability in the frequency of oligoarthritis, ranging from 25% to 65% [12]. In our study, asymmetric oligoarticular arthritis was the most common subtype, 20 (39.2%), followed by symmetric polyarthritis, 15 (29.4%), and DIP predominant arthritis, 11 (21.6%). Furthermore, several studies have identified polyarticular PsA as the most common subtype [13]. This difference may be attributed to the natural progression of oligoarthritis to polyarthritis over time. For instance, a longitudinal study conducted in Toronto found that 39% of oligoarthritis patients developed polyarthritis [12]. Additionally, previous studies utilizing ultrasound have reclassified oligoarticular patients as polyarticular due to the detection of subclinical disease with imaging [14].

Numerous studies have indicated an elevated risk of cardiovascular diseases in patients with PsA. This increased risk is attributed to a combination of traditional cardiovascular risk factors and the activity of the underlying disease. For example, a systematic review and meta-analysis of observational studies revealed that individuals with PsA face a 43% increased risk of cardiovascular diseases and a 55% increased risk of incidental cardiovascular events compared to the general population. This heightened risk was similar to that seen in patients with severe psoriasis [7]. In our study, cardiovascular comorbidities such as hypertension, type 2 diabetes mellitus, and hyperlipidemia were observed in 25 (47.2%) of PsA patients and 126 (35.6%) of those with psoriasis alone. Among PsA patients, type 2 diabetes mellitus and hypertension were the most common comorbidities, each affecting 16 (30.2%). Among psoriasis-only patients, type 2 diabetes mellitus was the most prevalent comorbidity at 83 (23.4%), while hypertension cases accounted for 68 (19.2%). Our findings are consistent with existing literature, which frequently reported a higher prevalence of hypertension in PsA patients compared to both the general population and individuals with psoriasis alone [15,16].

Psychiatric comorbidities are commonly observed in patients with PsA and are linked to an increased burden on the healthcare system [17]. In our study, depression and anxiety overall were more prevalent in PsA patients, 6 (11.3%), compared to those with psoriasis alone, 18 (5.1%). Depression was the most common psychiatric comorbidity in both groups, diagnosed in 5 (9.4%) of PsA patients and 11 (3.1%) of psoriasis-only patients, in keeping with previous studies [18,19]. Individuals with depression were 3.25 times more likely to be diagnosed with PsA than those without depression (OR = 3.25; 95% CI: 1.08-9.75). Importantly, logistic regression analysis identified psychiatric comorbidities as significant independent predictors of PsA, with an odds ratio of 4.5 (95% CI: 1.17-17.04). This association suggests that depression and anxiety may not only be consequences of PsA but may also play a role in its pathogenesis. Prior studies have proposed a bidirectional relationship, where psychological stress and inflammation may reinforce each other, potentially promoting the onset or exacerbation of autoimmune conditions [20]. Therefore, further research is needed to determine the role of early detection and treatment of psychiatric conditions in psoriasis patients in the prevention of PsA.

Another predictor of PsA through logistic regression analysis was family history. Despite only 9 (4.4%) of psoriasis patients having a family history of the disease, the model was statistically significant. Individuals with a family history of PsA had a 7.8-fold (β = 2.05) increased likelihood of developing the condition (OR = 7.8; 95% CI: 1.44-42.2). Therefore, incorporating family history into routine risk assessment tools may improve early identification of PsA. In addition, other predictors of PsA in the literature, including obesity, mechanical stress, and a history of infection, were not statistically significant in our analysis [21,22]. This outcome does not necessarily negate their clinical relevance, as sample size and population differences may affect statistical power.

Limitations

This study has several limitations. As a cross-sectional study, causality cannot be inferred. However, to examine potential risk factors for PsA, a nested case-control design was utilized within the cohort, allowing for analytical comparison between patients with and without PsA. The single-center design and relatively small sample size may limit generalizability. Furthermore, discrepancies in the number of patients across individual parameters reflect missing data from medical records. Although attempts were made to retrieve incomplete records by contacting patients via phone, not all were reachable. Despite this, we included all available data to preserve statistical power and minimize selection bias.

## Conclusions

This study provides valuable insights into PsA among psoriasis patients, revealing epidemiological information, key patterns, and associations. The prevalence of PsA in our cohort was consistent with Asian estimates, with females more frequently affected. Asymmetric oligoarticular arthritis emerged as the predominant subtype, followed by symmetric polyarthritis and distal interphalangeal joint involvement. Cardiovascular and psychiatric comorbidities, particularly depression, were more common in PsA patients compared to those with psoriasis alone. Additionally, a family history of PsA and psychiatric comorbidities was a strong predictor. These findings underscore the importance of early screening and a multidisciplinary approach to optimize patient outcomes. Future research involving larger, multicenter cohorts is recommended to validate these findings and further explore other risk factors and management strategies.
